# Oxide *versus* oxynitride cobalt and nickel thin-film electrocatalysts prepared by reactive sputtering for alkaline oxygen reduction reaction

**DOI:** 10.1039/d6ra02496c

**Published:** 2026-04-29

**Authors:** Aiman Hakim Supee, Shion Sugimoto, Yosuke Ishii, Shinji Kawasaki

**Affiliations:** a Department of Life Science and Applied Chemistry, Nagoya Institute of Technology Gokiso-cho, Showa-ku Nagoya 466-8555 Japan s.aimanhakim.571@stn.nitech.ac.jp yosuke.ishii@nitech.ac.jp kawasaki.shinji@nitech.ac.jp

## Abstract

Transition-metal oxynitrides are promising earth-abundant oxygen reduction reaction (ORR) catalysts, but conventional nitridation routes often limit control of nitrogen incorporation. Here, cobalt (Co)- and nickel (Ni)-based oxide and oxynitride were synthesized by reactive magnetron sputtering, producing uniform and adherent thin-film coatings on conductive substrates (Ti disk), and the ORR performance in alkaline O_2_-saturated 0.1M KOH aqueous solution was evaluated. Scanning electron microscopy (SEM) and X-ray diffraction (XRD) of CoO_1.6_N_0.12_ and NiO_1.14_N_0.18_ indicate amorphous films, while X-ray photoelectron spectroscopy (XPS) confirms the oxygen and nitrogen incorporation, and X-ray absorption near edge structure (XANES) Ni and Co K-edge shows a small positive edge shift upon nitridation. Relative to the corresponding oxides, the oxynitrides samples exhibited enhanced ORR activity and higher reaction electron number, with CoO_1.6_N_0.12_ showing consistently better performance (onset potential, *E*_onset_ = 0.82 V *vs.* RHE, reaction electron number, *n* ≈ 4.0) compared to the other samples. To demonstrate transferability to porous supports, CoO_*x*_N_*y*_ was sputtered onto single-walled carbon nanotubes (SWCNTs) achieving *E*_onset_ = 0.83 V and half-wave potential, *E*_1/2_ = 0.71 V *vs.* RHE with *n* ≈ 3.75 and ∼15% H_2_O_2_ yield. These results highlight reactive sputtering as a controllable route to fabricate oxide/oxynitride ORR electrodes and to upgrade carbon nanotubes-based cathode materials.

## Abstract 2

Reactive sputtering enables Co and Ni oxide/oxynitride thin films, where nitrogen incorporation enhances ORR activity, with Co oxynitride showing near four-electron selectivity.

## Graphical abstract

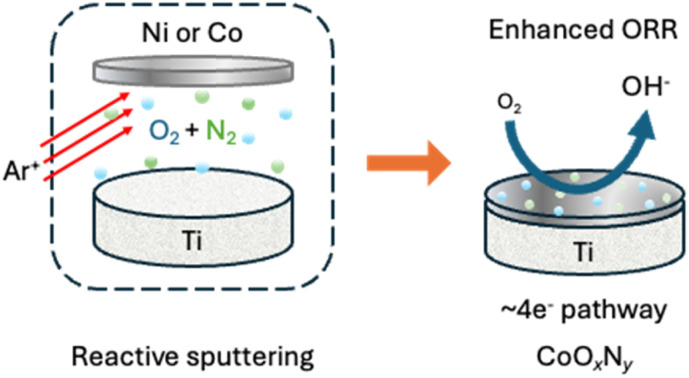

## 1 Introduction

Oxygen reduction reaction (ORR) kinetics at the cathode remain a key performance limitation in alkaline electrochemical energy conversion and storage technologies, which motivates continued development of more active catalysts. ORR activity is commonly benchmarked using rotating disk electrode (RDE) or rotating ring-disk electrode (RRDE) polarization measurements, where the onset potential and half-wave potential provide practical metrics for comparing catalytic performance under controlled mass-transport conditions. Pt-based catalysts are still the benchmark for ORR because of their high activity, but their cost and limited abundance hinder large-scale deployment. This has driven interest in earth-abundant alternatives such as transition-metal oxides, which offer low cost, yet typically exhibit lower ORR activity than Pt in pristine form and therefore, require strategies to enhance their catalytic performance.

In alkaline media, the ORR can proceed *via* either a direct 4e^−^ pathway, producing OH^−^, or a 2e^−^ pathway that generates peroxide intermediates (HO_2_^−^). The 4e^−^ pathway is generally preferred due to its higher efficiency and minimized formation of reactive peroxide species. The reaction pathway is strongly influenced by the catalyst's electronic structure and surface composition, which govern the adsorption and transformation of oxygenated intermediate such as OOH*, O*, or OH*. In transition-metal oxide systems, modification of the anion environment, such as nitrogen incorporation to form oxynitrides, can alter metal–oxygen bonding characteristics and thereby influence ORR activity and selectivity.

Among these, cobalt- and nickel-based oxides are widely studied as earth abundant electrocatalysts for the ORR in alkaline media and are often used as practical benchmark oxide platforms because their composition and surface chemistry can be systematically varied and evaluated under well-defined electrochemical conditions.^1,2,3^ Building on these oxide baselines, introducing nitrogen to form oxynitride-type compositions has been investigated as a strategy to tune electronic structure and local bonding environment, which can influence charge-transfer characteristics and the interaction strength of oxygenated intermediates relevant to ORR.^4^ Consequently, direct oxide-to-oxynitride comparisons under identical testing protocols are of interest for isolating the role of nitrogen incorporation in ORR activity, but achieving such comparisons in practice depends on synthesis routes that can control oxygen and nitrogen incorporation in a reproducible manner.

Conventional methods for incorporating nitrogen into transition metal oxides often rely on high temperature nitridation or ammonolysis of pre-formed oxides, and they frequently provide limited control over the resulting oxygen and nitrogen chemistry. The degree of nitrogen incorporation is highly sensitive to processing temperature, duration, and gas composition, which can lead to variability in stoichiometry and, in some cases, the formation of mixed oxide, oxynitride, and nitride phases.^5,6^ This motivates the use of alternative synthesis routes that offer more direct control over oxygen and nitrogen incorporation in the resulting films.

Reactive sputtering is a versatile approach for producing transition-metal oxide and oxynitride thin films by introducing reactive gases during sputter deposition. In mixed Ar/O_2_/N_2_ plasmas, nitrogen-containing oxide films have been directly deposited by reactive magnetron sputtering, demonstrating that oxynitride compositions can be formed without relying on high-temperature post-nitridation steps.^7^ Moreover, independent control of the O_2_ and N_2_ feed conditions (*e.g.*, partial pressures/flow ratios or pulsing parameters) can switch the deposition between oxidised and nitride sputtering regimes, enabling systematic tuning of oxide-to-oxynitride character in the deposited films.^8^ This provides a practical alternative to conventional high-temperature nitridation or wet-chemical doping routes that often suffer from poor reproducibility and limited control of nitrogen incorporation. Importantly, magnetron sputtering can produce uniform, adherent films with defined thickness on conductive substrates.^9^ This facilitates the preparation of model electrodes for direct, like-for-like electrocatalytic comparisons of oxide and oxynitride materials under standardized testing conditions.

In this study, reactive sputtering is employed as a controlled and versatile synthesis platform to systematically investigate the influence of nitrogen incorporation on ORR activity in transition-metal oxide systems. While nitrogen incorporation has been widely reported to enhance ORR performance, direct comparisons between oxide and oxynitride phases remain challenging due to variations in synthesis methods, morphology, and compositions. Reactive sputtering offers a straightforward and controllable approach for incorporating oxygen and nitrogen during film growth without requiring post-treatment steps. Here, Co- and Ni-based oxide and oxynitride thin films (CoO_*x*_, CoO_*x*_N_*y*_, NiO_*x*_, and NiO_*x*_N_*y*_) are prepared under comparable deposition conditions to enable direct, like-for-like comparisons. By minimizing variations in thin film morphology and thickness, this approach enables clearer isolation of the effect of nitrogen incorporation on electrocatalytic performance. ORR activity is evaluated in O_2_-saturated 0.1M KOH using rotating ring-disk electrode (RRDE) measurements, with performance benchmarked using onset and half-wave potentials. This study provides insight into how nitrogen incorporation and transition metal identity (Co *vs.* Ni) influence ORR activity under well-defined conditions.

## 2 Experimental

### 2.1 Materials and reagents

Nickel sputtering targets (99.9% purity, 50.8 mm diameter, 2 mm thickness) were purchased from Kojundo Chemical Laboratory. A cobalt plate (99.9% purity, 0.40 mm thickness) obtained from Nilaco was cut into a circular target with a diameter of 50.8 mm prior to use. Titanium substrates were used as conductive supports for thin film deposition. Titanium foil (99.5% purity, 0.05 mm thickness) and titanium rods (99.5% purity) were supplied by Nilaco. The titanium rods were machined into cylindrical electrodes with diameter of 4.05 mm and a length of 4 mm, followed by mechanical polishing prior to use. Single-walled carbon nanotubes (SWCNTs, Tuball 01RW03, average diameter ∼1.6 nm, surface area >800 m^2^ g^−1^) were obtained from OCSiAl and used as a high surface-area support for catalyst deposition. Ethanol (analytical grade) was used as the solvent for catalyst ink preparation, and a Nafion solution (5 wt%) was used as a binder. Potassium hydroxide (KOH, analytical grade) was used to prepare the electrolyte solution for electrochemical measurements. All chemicals were used as received without further purification.

### 2.2 Instrumental

**Fig. 1:**
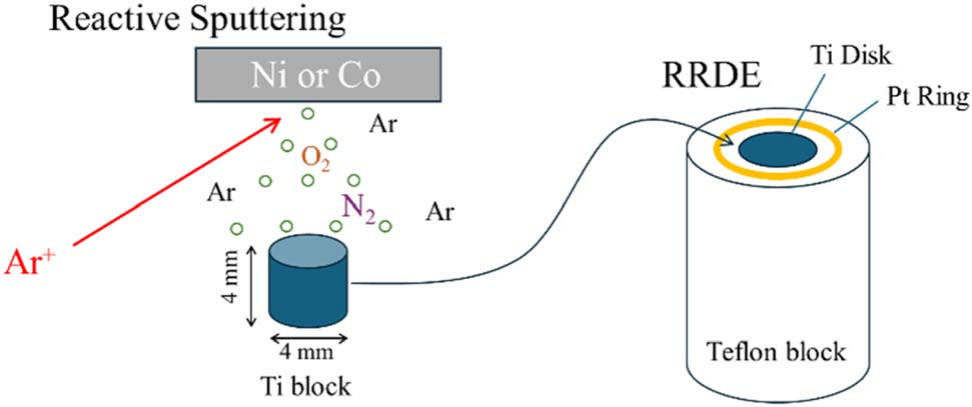
RRDE setup for the evaluation of ORR performance for the deposited transition metal oxides/oxynitrides on the Ti block.

Co and Ni thin films were deposited onto cylindrical Ti substrates using a magnetron sputtering system (VTR-151M/SRF, ULVAC). Reactive sputtering was carried out in Ar-based atmosphere containing O_2_ and N_2_. The total chamber pressure (Ar + O_2_ + N_2_) was maintained at 0.5 Pa, with sputtering power of 50 W and the substrate kept at room temperature. Each deposition consisted of a 20 min pre-sputter followed by a 60 min deposition. The nitrogen incorporation was controlled by adjusting the O_2_ and N_2_ partial pressures. The sputtered Ti cylinders were embedded in a Teflon holder for electrochemical measurements (Fig. 1). X-ray Photoelectron Spectroscopy (XPS), X-ray Diffraction (XRD), X-ray Absorption Near Edge Structure (XANES), and Scanning Electron Microscopy (SEM) experiments were also performed.

### 2.3 Electrochemical experiments

ORR electrochemical measurements were performed using a disk-replaceable rotating ring-disk electrode (RRDE; DRE-PTR, BAS) setup. A Pt wire and a Hg/HgO electrode were used as the counter and reference electrodes, respectively. The electrolyte was 0.1 mol L^−1^ KOH aqueous solution, which was saturated with O_2_ by bubbling for 30 min prior to each measurement. During the experiment, the ring electrode was held at +0.6 V *vs.* Hg/HgO. Linear Sweep Voltammetry (LSV) on the disk electrode was recorded from +0.4 V to −0.8 V *vs.* Hg/HgO at a scan rate of 10 mV s^−1^ and a rotation rate of 1600 rpm. All potentials were converted to the reversible hydrogen electrode (RHE) scale using

1 *E*_RHE_ = *E*_Hg/HgO_ + *E*^0^_Hg/HgO_ + 0.0581 × pH

where, *E*^0^_Hg/HgO_ is the standard potential of the Hg/HgO reference electrode *versus* SHE for 1M NaOH aqueous solution. The pH of 0.1M KOH aqueous solution was taken as ∼13. The reaction electron number, *n* and peroxide yield were calculated from the RRDE disk current *I*_D_ and ring current *I*_R_ using the collection efficiency *N* (taken as 0.37) of the RRDE.2
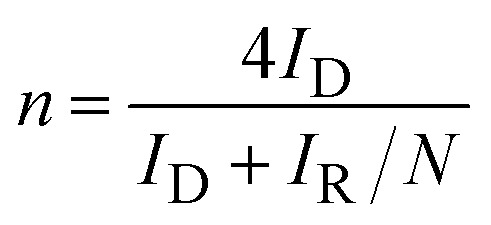
3
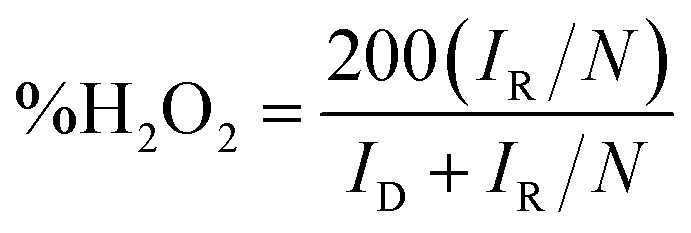


Separately, pristine SWCNT electrodes were prepared by dispersing 1 mg of SWCNT in 950 µL of ethanol and 50 µL Nafion dispersion, followed by stirring and sonication. 3.85 µL of the ink was drop-cast onto the Ti electrode and dried under ambient conditions prior to sputtering. The CoO_*x*_N_*y*_-CNT electrode was prepared by depositing CoO_*x*_N_*y*_ onto the SWCNT-coated disk using the same reactive magnetron sputtering system under an Ar/O_2_/N_2_ atmosphere at room temperature. The resulting electrodes were used directly for RRDE measurements under the same conditions as described above.

## 3 Results & discussion

### 3.1 Characterization of catalytic system

**Fig. 2:**
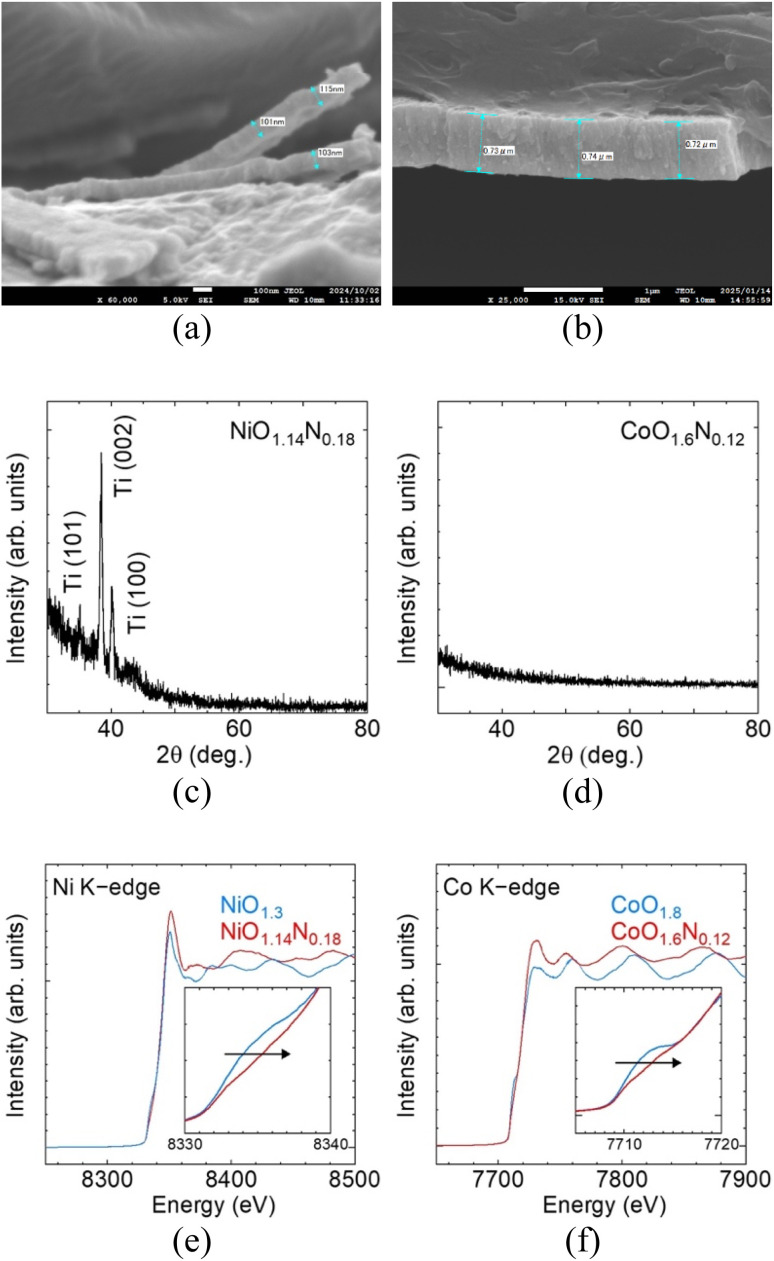
Cross-sectional SEM images of (a) NiO_1.14_N_0.18_, (b) CoO_1.6_N_0.12_, thin-film XRD patterns of (c) NiO_1.14_N_0.18_, (d) CoO_1.6_N_0.12_, and XANES spectra at the (e) Ni K-edge and (f) Co K-edge for oxide and oxynitride films.

SEM, thin-film XRD, and XANES characterisation of the sputter-deposited films are summarised in Fig. 2. Despite identical deposition time and reactive-gas atmosphere, the resulting film thickness differed between Ni and Co. NiO_*x*_N_*y*_ formed a thin layer of ∼100 nm while CoO_*x*_N_*y*_ grew to ∼700 nm (Fig. 2a and b). Thin-film XRD shows no discernible reflections attributable to the deposited layers (Ti substrate peaks are visible for the thinner Ni film), indicating that both deposits are amorphous or highly disordered (Fig. 2c and d). Elemental analysis by XPS (Table S1) confirms incorporation of O and N for films prepared under N_2_, yielding compositions (normalised to metal = 1) of NiO_1.14_N_0.18_ and CoO_1.6_N_0.12_, compared with NiO_1.3_ and CoO_1.8_ in the absence of N_2_. The NiO_1.3_ composition is close to stoichiometric NiO, while CoO_1.8_ is slightly more O-rich than the Co_3_O_4_ stoichiometry. Ni and Co K-edge XANES spectra exhibit small positive shift of the absorption edge upon nitrogen incorporation (Fig. 2e and f), consistent with a change in the local electronic structure, such as a higher average metal oxidation state.^10^

**Fig. 3:**
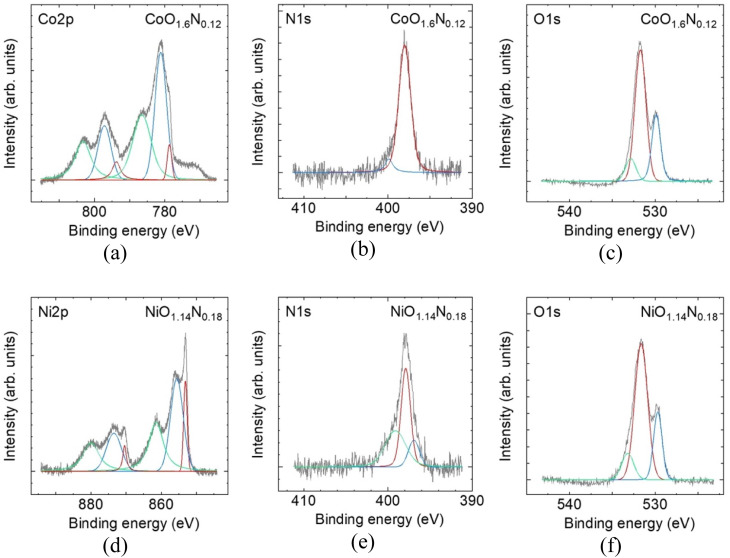
XPS spectra of reactive-sputtered Co and Ni oxynitride films. (a–c) for CoO_1.6_N_0.12_ of Co2p, N1s, and O 1s, respectively and (d–f) for NiO_1.14_N_0.18_ for Ni2p, N 1s, and O 1s, respectively. Black line shows experimental data and the coloured curves indicate fitted components.

XPS was performed to examine surface chemical states and verify nitrogen incorporation in the sputtered films, and the results can be summarized in Fig. 3. While the XANES edge shift suggests that nitrogen incorporation is accompanied by a change in the metal electronic environment, detailed oxidation-state assignment from the Ni and Co core-level spectra is not straightforward because Ni^2+^/Ni^3+^ and Co^2+^/Co^3+^ components overlap in the present Co- and Ni-based films. Nevertheless, a low-binding energy contribution is observed for both metals, suggesting the presence of residual metallic species.

In contrast, nitrogen incorporation is clearly evidenced in the N 1s region, where a peak is detected only for films deposited with a finite N_2_ partial pressure, whereas no N 1s signal is detected for the oxide films prepared without N_2_. The N 1s binding energy of ∼397 eV is consistent with nitrogen in a metal-N bonding environment commonly assigned to nitride or oxynitride-type species, rather than oxidized or adsorbed nitrogen.^11,12^ This observation provides a plausible explanation for the positive XANES shift, since partial substitution of O^2−^ by N^3−^ requires charge compensation and can contribute to a higher average metal oxidation state or modified local coordination around the metal centres.

The O 1s spectra for both Co and Ni films can be fitted with two components. The lower binding energy (∼530 eV) is commonly assigned to lattice oxygen in the metal oxide network. The higher binding energy (∼532 eV) component is commonly attributed to oxygen in defect related or low coordination environments that may arise from oxygen vacancies or surface reconstruction and surface functional groups.^13,14^ The overall O 1s line shape changes only weakly after nitridation for both Co and Ni. This suggests that the oxide films are already highly disordered which is consistent with room temperature reactive sputtering where limited atomic mobility can hinder lattice stabilisation and produce defect rich amorphous structure. As a result, any additional defect signatures introduced by nitrogen incorporation may be masked which explains the minimal change observed in the O 1s profile.

### 3.2 Electrochemical properties of Co-and Ni-systems

**Fig. 4:**
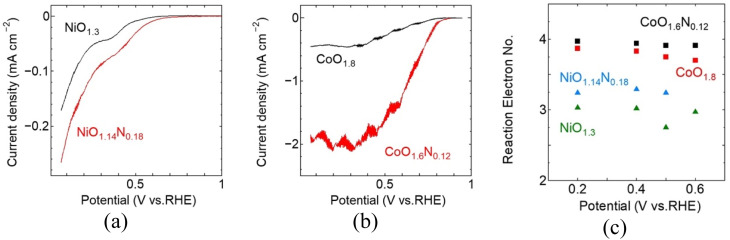
RRDE ORR performance of reactive-sputtered Ni and Co oxide and oxynitride films in O2-saturated 0.1M KOH for (a) NiO_1.3_ and NiO_1.14_N_0.18_, and (b) CoO_1.8_ and CoO_1.6_N_0.12_. (c) Reaction electron number calculated from disk and ring current as a function of potential for the corresponding films.

The RRDE-LSV results for the sputter-deposited Ni and Co films are shown in Fig. 4. Under identical measurement conditions, the Co-based films exhibit more favourable ORR polarisation behaviour than the Ni-based films. Because the current density was normalised to geometric area and the Co films were substantially thicker than the Ni films under identical sputtering conditions, the Co–Ni performance difference likely reflects both intrinsic catalytic effects and differences in catalyst loading. The intrinsic differences in Co- and Ni-based active site can lead to different adsorption strengths and selectivity for key oxygenated intermediates.^15^ More favourable intermediate binding on Co sites could potentially lower the kinetic barriers for ORR steps and promote a more direct 4e^−^ pathway. A similar trend has been reported for Co- and Ni-based oxygen electrocatalysis in alkaline media, where Co-containing samples often show more favourable ORR activity than Ni-based samples.^16^ For both metals, nitrogen incorporation shifts the ORR curves positively relative to the corresponding oxides, with the *E*_onset_ increases from 0.51 to 0.60 V for NiO_1.3_ and NiO_1.14_N_0.18_, respectively, and from 0.78 to 0.82 V for CoO_1.8_ and CoO_1.6_N_0.12_, respectively. In both cases, the cathodic current density also increases after nitrogen incorporation, demonstrating improved ORR activity. A comparison of the ORR performance with previously reported catalysts is provided in Table S1.

### 3.3 ORR efficiency for Co- and Ni-systems

To further evaluate ORR efficiency and reaction pathway behaviour, RRDE measurements were used to estimate the electron transfer number (*n*) from the disk and ring currents. Nitrogen incorporation leads to an increase in *n* for both metal systems reaching ∼3.2 for NiO_1.14_N_0.18_ and approaching ∼4.0 for CoO_1.6_N_0.12_. This indicates a shift toward a more efficient 4e-reduction pathway with reduced peroxide formation compared to the corresponding oxide samples. Overall, under the present conditions, the Co-based system shows a more pronounced improvement based on *E*_onset_ and *n*, upon nitrogen incorporation compared to the Ni-based system, suggesting that Co may be a more favourable host matrix for nitrogen doping, although differences in film thickness may also contribute to the observed trends.

Nitrogen incorporation is expected to improve ORR performance by modifying the metal-anion framework and the surface chemistry of the deposited films. Partial substitution of O^2−^ by N^3−^ perturbs the local bonding environment and electronic structure around the metal centres, which can shift the adsorption strength of key oxygenated intermediates toward more favourable values for ORR.^17,18,19^ This electronic tuning can lower the kinetic barrier for O_2_ activation and subsequent reduction steps and can reduce the tendency toward peroxide formation, which is consistent with the increase in reaction electron number observed in oxynitride samples.^20^ In addition, nitrogen incorporation can also promote local structural disorder within these sputtered films and increases the defect-related environments that provide favourable binding sites for O_2_ and reaction intermediates.^21^ Therefore, although the present data do not directly identify the active sites, the combined improvements in polarisation behaviour and selectivity support the conclusion that nitridation enhances ORR activity through coupled electronic-structure and defect-chemistry effects in the oxynitride films. While direct quantification of defect density was not performed, the higher ORR activity and *n* of CoO_*x*_N_*y*_ suggest more favourable active environments, potentially related to higher structural disorder and local bonding introduced during nitridation. A definitive comparison of defect density would require further characterization beyond the scope of this work.

**Fig. 5:**
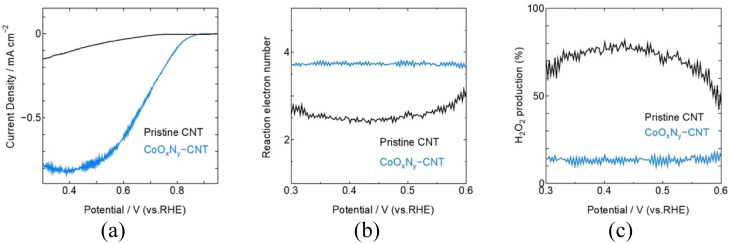
RRDE evaluation of CoO_*x*_N_*y*_ sputtered onto CNTs in O_2_-saturated 0.1M aqueous KOH solution at 1600 rpm, 10 mV s^−1^ scan rate. (a) LSV disk polarization curve, (b) reaction electron number, (c) H_2_O_2_ yield calculated from the disk and ring potential.

Considering practical application, CoO_*x*_N_*y*_ was sputter-deposited onto high-surface SWCNTs to demonstrate that this deposition strategy is applicable to porous carbon supports. The RRDE evaluation of CNT coated with sputtered CoO_*x*_N_*y*_ under identical reactive conditions as CoO_1.6_N_0.12_, shows that reactive sputtering enhances ORR activity while maintaining a near 4e-reduction pathway (Fig. 5). Compared with pristine CNT, which displayed a limited ORR response, CoO_*x*_N_*y*_-CNT exhibit markedly improved polarization behaviour, achieving *E*_onset_ = 0.83 V and*E*_1/2_ = 0.71 V *vs.* RHE. The selectivity analysis derived from disk and ring currents gives an average *n* ≈ 3.75, consistent with a modest peroxide yield of approximately 15% across the measured potential range (Fig. S8). Together, these results indicate that CoO_*x*_N_*y*_ remains effective when translated from model thin films to a high-surface area CNT scaffold, delivering improved ORR activity with limited H_2_O_2_ formation.

## 4 Conclusion

Co- and Ni-based oxide and oxynitride thin films (CoO_1.8_, CoO_1.6_N_0.12_, NiO_1.3_, NiO_1.14_N_0.18_) were successfully prepared by reactive magnetron sputtering on Ti substrates and evaluated by RRDE measurements for alkaline ORR in O_2_-saturated 0.1M KOH aqueous solution. Nitrogen incorporation shifted the XANES absorption edge to higher energy for both metals, consistent with a modified metal electronic structure, and improved ORR performance relative to the corresponding oxides. RRDE analysis showed increased reaction electron number upon nitridation, with CoO_1.6_N_0.12_ exhibiting the most 4e^−^ selective behaviour (*n* ≈ 4.0) and *E*_onset_ = 0.82 V *vs.* RHE. Extending the method to high-surface area supports, CoO_*x*_N_*y*_ sputtered onto SWCNTs delivered *E*_onset_ = 0.83 V, and *E*_1/2_ = 0.71 V *vs.* RHE with *n* = 3.75 and ∼15% H_2_O_2_ yield, demonstrating improved ORR activity with limited peroxide formation. Overall, reactive sputtering offers a versatile route to tune oxide/oxynitride electrocatalyst electrodes and translate oxynitride chemistry to SWCNT-based cathode architectures.

## Conflicts of interest

There are no conflicts of interest to declare.

## Supplementary Material

RA-016-D6RA02496C-s001

## Data Availability

The data supporting this article have been included as part of the supplementary information (SI). All the references cited in the SI have been listed in the article's reference list.^22–24^ Supplementary information: EDS elemental analysis, XPS elemental composition, XRD pattern, XANES spectra at Ni K-edges and Co K-edges. See DOI: https://doi.org/10.1039/d6ra02496c.
