# Supplementary material for: Oxide versus oxynitride cobalt and nickel thin-film electrocatalysts prepared by reactive sputtering for alkaline oxygen reduction reaction
Source: RSC Adv. 2026 Apr 29;16(25):22368–74. doi: 10.1039/d6ra02496c (PMC13127378; doi:10.1039/d6ra02496c)
Supplement: RA-016-D6RA02496C-s001 [file RA-016-D6RA02496C-s001.pdf]

Supplementary Information (SI) for RSC Advances.
This journal is © The Royal Society of Chemistry 2026

Oxide versus oxynitride Co and Ni thin-film electrocatalysts prepared by reactive sputtering for alkaline ORR

*Aiman Hakim Supee, Shion Sugimoto, *Yosuke Ishii, *Shinji Kawasaki

Department of Life Science and Applied Chemistry, Nagoya Institute of Technology
Gokiso-cho, Showa-ku, Nagoya, 466-8555, Japan

Corresponding Author

Email: s.aimanhakim.571@stn.nitech.ac.jp , yosuke.ishii@nitech.ac.jp, kawasaki.shinji@nitech.ac.jp

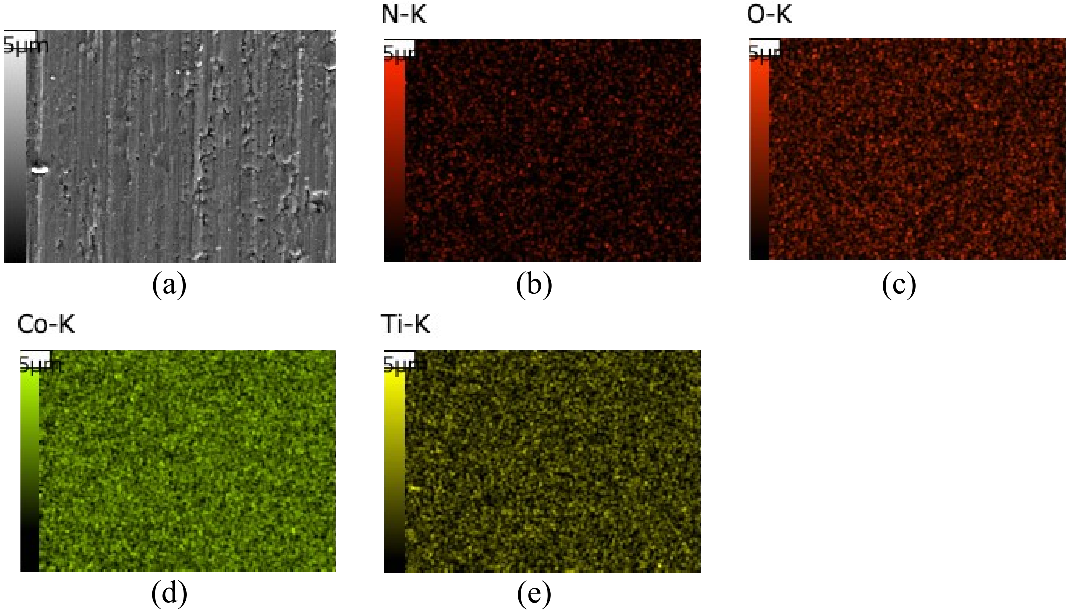

Figure S1 EDS elemental analysis of $CoO_{1.6}N_{0.12}$ sputtered on Ti block. Spectra were acquired from the film region shown in the (a) SEM image for (b) N, (c) O, (d) Co, and (e) Ti

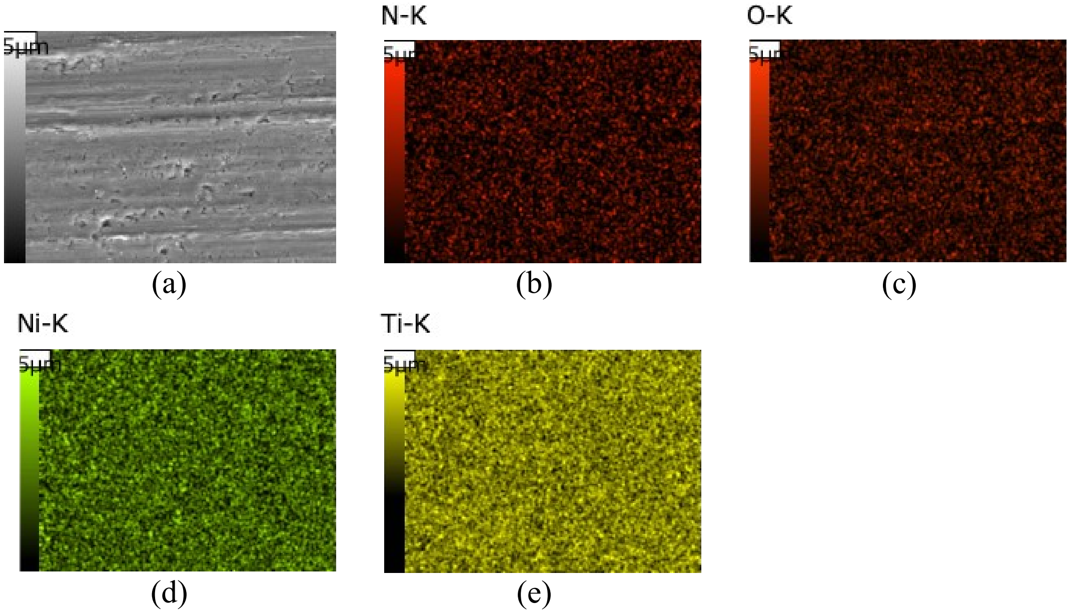

Figure S2 EDS elemental analysis of $NiO_{1.14}N_{0.18}$ sputtered on Ti block. Spectra were acquired from the film region shown in the (a) SEM image for (b) N, (c) O, (d) Ni, and (e) Ti

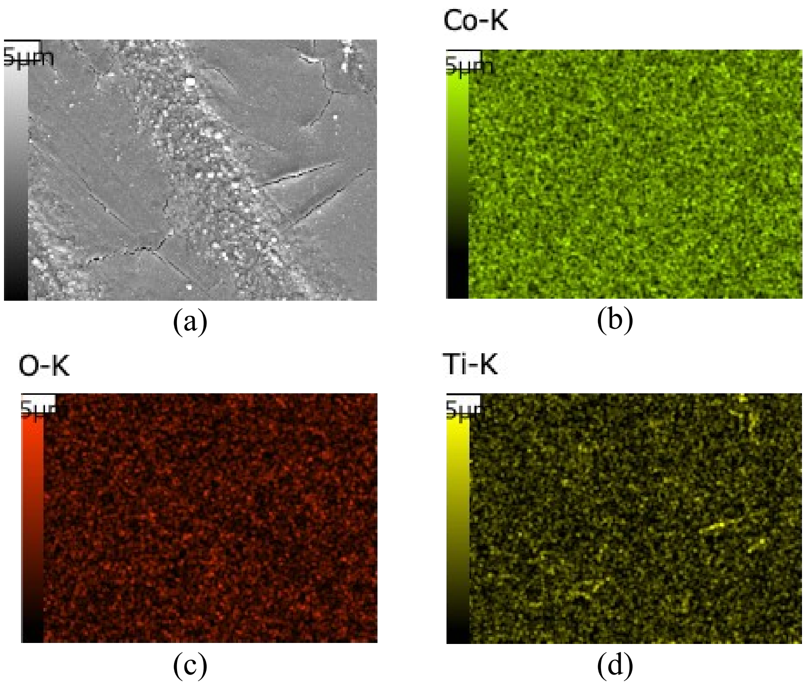

Figure S3 EDS elemental analysis of $CoO_{1.8}$ sputtered on Ti block. Spectra were acquired from the film region shown in the (a) SEM image for (b) Co, (c) O, and (d) Ti

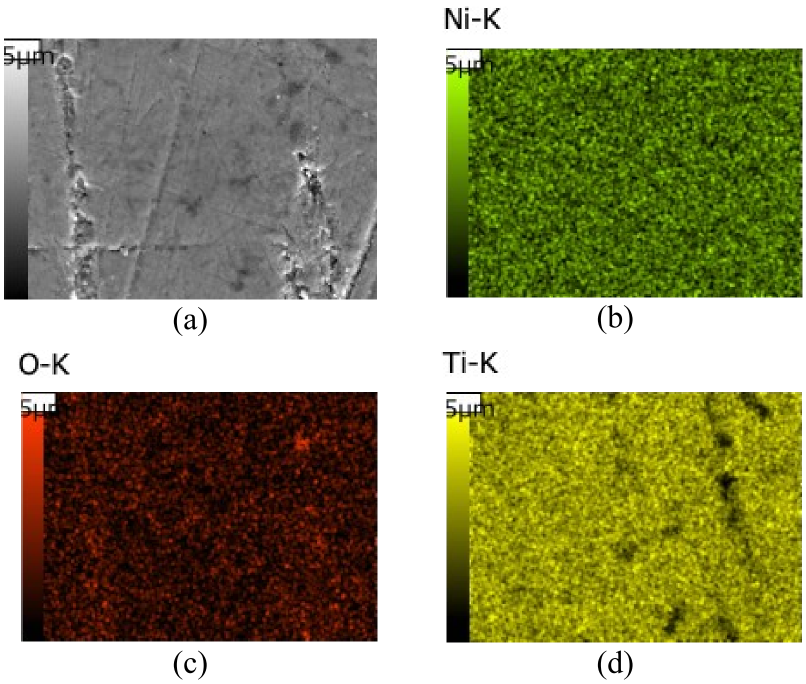

Figure S4 EDS elemental analysis of $NiO_{1.3}$ sputtered on Ti block. Spectra were acquired from the film region shown in the (a) SEM image for (b) Ni, (c) O, and (d) Ti

Table S1 Elemental composition determined from XPS analysis for the sputtered sample

Sample	Atomic concentration (%)				
	Co2p	Ni2p	O1s	N1s	C1s
$CoO_{1.6}N_{0.12}$	27.61	-	45.25	3.45	23.69
$NiO_{1.14}N_{0.18}$	-	31.86	36.29	5.75	26.10
$CoO_{1.8}$	25.75	-	47.04	0.32	26.89
$NiO_{1.3}$	-	31.29	40.58	0.60	27.53

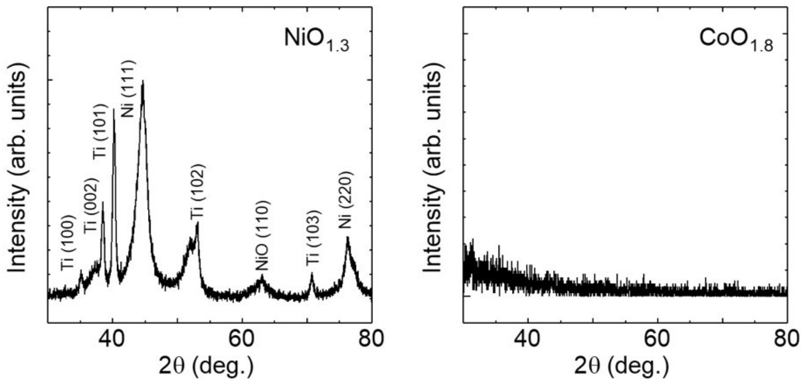

(a) (b)

Figure S5 Thin film XRD pattern for (a) $NiO_{1.3}$ and (b) $CoO_{1.8}$

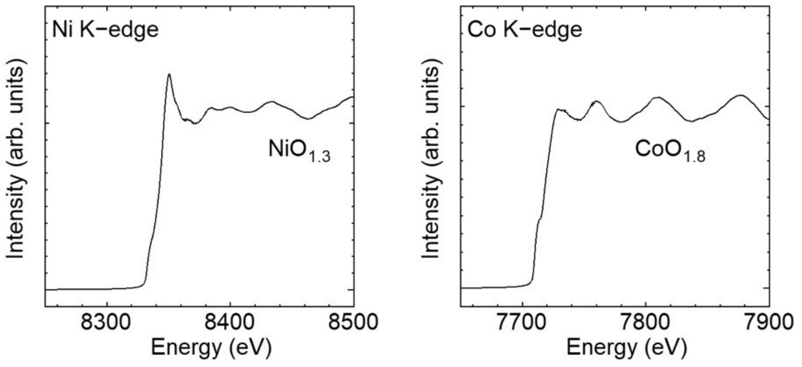

(a) (b)

Figure S6 XANES spectra at Ni K-edges for (a) $NiO_{1.3}$ and Co K-edge for (b) $CoO_{1.8}$

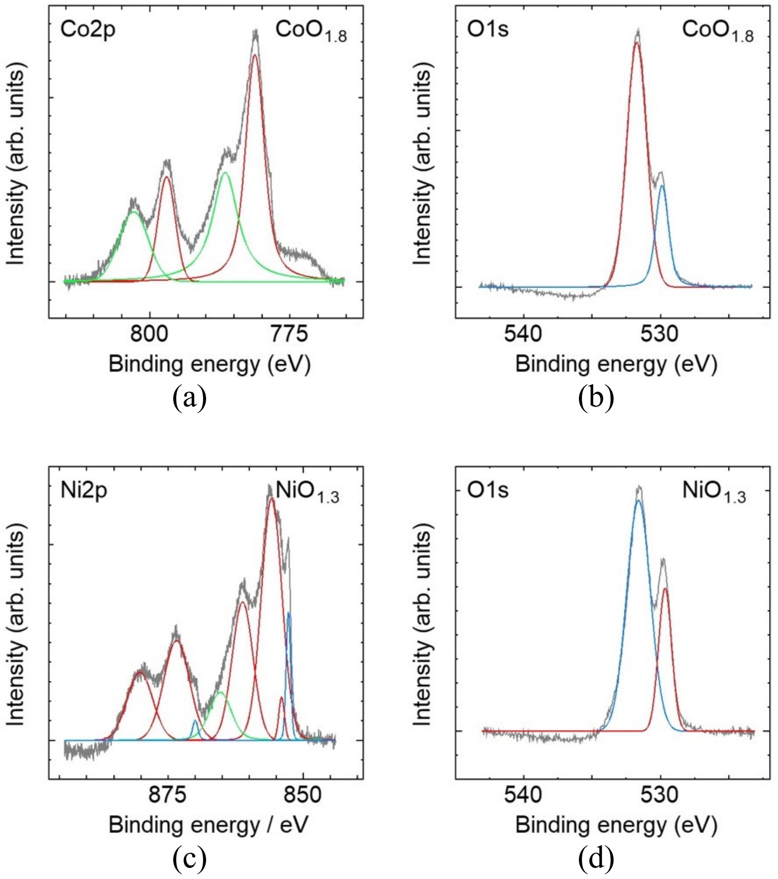

Figure S7 XPS spectra of reactive-sputtered Ni- and Co-based films. Shown are the (a) Co2p and (b) O1s for $CoO_{1.8}$ and (c) Ni2p and (d) O1s for $NiO_{1.3}$

Table S2 Performance comparison with other catalyst

Catalyst	ORR onset potential (V vs RHE)	Electron transfer number	Electrolyte	Ref.
rGO/(Ni^{2+}-THPP/Co^{2+}-THPP)$_8$	0.84	3.83	0.1M KOH aq.	[22]
NiN_2Ge_2	0.92	-	N/A	[23]
Pd@CoO_x/NC1	1.02	4.02	0.1M KOH aq.	[2]
Co_3O_4/CIMP-MW	0.83	3.71	0.1M NaOH aq.	[24]
CoO_xN_y on Ti disk	0.82	4.0	0.1M KOH aq.	This work
CoO_xN_y/SWCNT	0.83	3.75	0.1M KOH aq.	This work
